# Inhibition of dengue NS2B-NS3 protease and viral replication in Vero cells by recombinant retrocyclin-1

**DOI:** 10.1186/1471-2334-12-314

**Published:** 2012-11-21

**Authors:** Hussin A Rothan, Heh Choon Han, Thamil Selvee Ramasamy, Shatrah Othman, Noorsaadah Abd Rahman, Rohana Yusof

**Affiliations:** 1 Department of Molecular Medicine, Faculty of Medicine, University of Malaya, 50603, Kuala Lumpur, Malaysia; 2 Department of Pharmacy, Faculty of Medicine, University of Malaya, 50603, Kuala Lumpur, Malaysia; 3 Department of Chemistry, Faculty of Science, University of Malaya, 50603, Kuala Lumpur, Malaysia

**Keywords:** Retrocyclin-1, Recombinant peptides, Dengue virus, NS2B-NS3 protease, Protease activity, Viral inhibition

## Abstract

**Background:**

Global resurgence of dengue virus infections in many of the tropical and subtropical countries is a major concern. Therefore, there is an urgent need for the development of successful drugs that are both economical and offer a long-lasting protection. The viral NS2B-NS3 serine protease (NS2B-NS3pro) is a promising target for the development of drug-like inhibitors, which are not available at the moment. In this study, we report retrocyclin-1 (RC-1) production in *E. coli* as a recombinant peptide to test against dengue NS2B-NS3pro.

**Methods:**

Dengue NS2B-NS3pro was produced as a recombinant single chain protein in *E. coli* and purified by Ni^+^ affinity chromatography. The RC-1 peptide was produced in *E. coli* and the tri-disulphide bonds were reformed in a diluted alkaline environment. Protease assay was performed using a fluorogenic peptide substrate and measured by fluorescence spectrometry. Real-time PCR was used for quantification of dengue serotype 2 (DENV-2) viral RNA produced in Vero cells.

**Results:**

The RC-1 peptide inhibited the activity of recombinant NS2B-NS3pro with different values at 50% inhibitory concentration (IC_50_) which are temperature dependent (28°C, 46.1 ± 1.7 μM; 37°C, 21.4 ± 1.6 μM; 40°C, 14.1 ± 1.2 μM). The presence of RC-1 significantly reduced viral replication in Vero cells infected with DENV-2 at simultaneous treatment after 48 hrs (70%) and 75 hrs (85%). Furthermore, moderate reduction in viral replication was observed at pre-treatment mode after 48 hrs (40%) and 72 hrs (38%) and post-treatment at 48 hrs (30%) and 72 hrs (45%).

**Conclusion:**

Recombinant RC-1 inhibits DENV-2 replication in Vero cells by interfering with the activity of its serine protease. Thus, we propose that recombinant RC-1 is a potent, cost-effective dengue virus inhibitor. Therefore, it is suitable to consider RC-1 as a new candidate for drug development against dengue infection.

## Background

Dengue is an acute febrile viral disease with hundreds of millions of infections occurring each year and more than half of the world population are now at risk [1]. The virus uses host cell ribosomes to translate its genomic RNA to full length precursor polyprotein. Subsequently, proteolytic cleavage of the polyprotein results in formation of structural and non-structural proteins [2]. It has been found that one of the most important non-structural proteins is the NS3 protein. It has a trypsin-like serine protease domain (NS3pro) located within the N-terminal 180 amino acid residues [3,4]. However, the activity of NS3pro depends on the interaction with its cofactor NS2B (47 amino acids) to form NS2B-NS3pro complex [5]. The importance of NS2B-NS3pro comes from its ability to cleave at various regions in the viral polyprotein [6-11]. It has been found that the disruption of NS2B-NS3pro function inhibits viral replication [12]. Therefore, the NS2B-NS3pro is considered as a potential target for antiviral drug design [13].

At present, there are neither vaccines nor other treatments available to prevent or cure this disease [14]. These facts emphasize the need for a better understanding of the mechanism of viral infection in order to combat this disease. Recently, computational studies indicated that disulphide cyclic peptides have potential to inhibit dengue NS2B-NS3pro [15,16]. In this study, our aim was to investigate the potential inhibition of retrocylin-1 (RC-1), a disulphide cyclic peptide, against dengue NS2B-NS3pro. It has been known that RC-1 (GICRCICGRGICRCICGR) is one of theta-defensins analogues which are circular, cationic, tri-disulphide bonded peptides with beta–sheet structure that can protect human peripheral blood lymphocytes from infection by HIV-1 strains [17]. This peptide is encoded in the human genome by a theta-defensins pseudogene [18]. However, it has only been found to play important roles in the innate host defense in the white blood cells of macaques, baboons and orang-utans [19,20]. Recent attempts have been made to confirm that human cells have the ability to produce correctly folded retrocyclins using aminoglycosides to read-through the premature termination codon found in the mRNA transcripts [21]. In our study, we successfully produced RC-1 in *E. coli* as a recombinant peptide. The soluble recombinant peptide was tagged with six histidine residues for metal column purification. To reform tri-disulphide bonds in the correct positions, the mis-folded peptide was refolded in alkaline and diluted environment. Recombinant RC-1 exhibited significant inhibitory potential against dengue NS2B-NS3pro and reduced viral replication in Vero cells. We propose that the recombinant RC-1 could be considered as a candidate therapeutic peptide against dengue virus infection.

## Methods

Mosquito cell lines C6/36 was expanded to 80% confluence and infected with DENV-2 viruses (Isolate D2MY04-32618) and incubated at 32°C for 3 days. To amplify the cDNA fragment of NS2B-NS3pro sequence, DENV-2 infected cells were harvested and viral RNA was extracted using viral RNA Extraction Kit (Promega, USA) based on to the manufacturer’s instructions. The cDNA fragment of *NS2B-NS3pro* was generated using the NS2BF (5’-ATACTGA*GGATCC*GCCGATTTGGAACTG-3’) and NS3R (5’-ATTGATC*AAGCTT*AAGCTTCAATTTTCT-3’) which was then used as template to amplify NS2B (amino acids 49–95) and NS3pro (amino acids 1–180) by PCR reactions.

### Construction of plasmids

#### pQE30-NS2B (G4-T-G4) NS3pro

To produce a single chain *NS2B (G*_*4*_*-T-G*_*4*_*) NS3pro*, the *NS2B* fragments were amplified separately by PCR using the primer pairs NS2BF and NS2BlikR (5’-ATACTGAGGATCCGCCGATTTGGAACTG-3’, 5-ACCTACTA*GGTACCTCCTCCACC*CAGTGTCTGTTCTTC-3’) while *NS3pro* was amplified using NS3likF and NS3R primers (5-ATCTATA*GGTACCGGCGGTGGAGGT*GCTGGAGTATTGTGG-3’, 5’-AGCATAAGCTTAAGCTTCAATTTTCT-3’). The linker sequence and *Kpn*I restriction sites were added to NS2BlikR and NS3likF primers. The PCR products were subsequently digested with *BamH*I and *Kpn*I And the purified fragments were cloned into pQE30 plasmid downstream of the 6XHis tag (all restriction sites are underlined).

#### pET 303/CT His –RC-1

The sense and antisense DNA fragments of 102 bases including the sequence of cleaved *Xba*I and *Xho*I restriction sites were chemically synthesized and hybridized. The double stranded DNA fragment was cloned directly into pET 303/CT His vector and transformed into *E. coli.*

### Protein production in *E. coli*

The *Escherichia coli* XL1-Blue strain (Promega, USA) was transformed with pQE30-*NS2B (G4-T-G4) NS3pro* and *pET-RC-1*. The recombinant *E. coli* was inoculated in Luria-Bertani liquid medium (1% tryptone, 1% NaCl, 0.5% yeast extract w/v, pH 7.0) supplemented with 100 mg/L ampicillin and cultured overnight at 37°C. In general, 10 ml of overnight-grown culture was added to 1000 ml of culture medium and incubated with shaking at 37°C until the optical density at 600 nm reached 0.5. Isopropylthio-β-D-galactoside (IPTG) was then added to the culture medium at a final concentration of 0.5 mM to induce protein expression and further incubation was applied for another 5 hrs at 37°C in a shaking incubator. Bacterial cells were harvested by centrifugation at 4000 rpm for 15 min at 4°C.

### Protein purification

The recombinant NS2B-NS3pro and RC-1 were produced as soluble proteins and purified using His GraviTrap^TM^ Flow precharged Ni Sepharose^TM^ 6 Fast column (Amersham Biosciences, USA) according to the manufacturer’s instructions. In brief, the column was equilibrated with phosphate buffer (20 mM sodium phosphate buffer and 500 mM NaCl, pH 7.4). The sample was loaded into the column and the column was washed with binding buffer (phosphate buffer containing 20 mM imidazole, pH 7.4). The recombinant protein was eluted with elution buffer (phosphate buffer containing 200 mM imidazole, pH 7.4).

### Refolding of recombinant RC-1

The mis-folded recombinant RC-1 was refolded as previously described [22]. In brief, urea was added to the purified RC-1 to a final concentration of 2 M and the pH was adjusted to 12.5. Then, disulphide bonds were reduced by 5 mM of β-mercaptoethanol and incubated at room temperature for 30 min with stirring. The denatured and reduced RC-1 was diluted to a final concentration of 0.01 mg/ml with ice-cold refolding buffer (100 mM Tris–HCl, 1 mM EDTA, 10% glycerol, 250 mM L-Arginine, 1 mM reduced Glutathione and 0.5 mM oxidized Glutathione (pH 12.5)). Protein sample was loaded into a dialysis tube and the dialysis was carried out overnight against 200 volumes of 100 mM Tris–HCl, pH 10 to eliminate the residuals of urea and β-mercaptoethanol. Overnight incubation of peptide samples at 4°C was performed before the refolded RC-1 was concentrated to 1 mg/ml by Vivaspin concentrating 50 ml tubes with a 3,000 MW cut-off membrane (Sartorius, Germany). The pH was adjusted to 8.0 and the linker and 6XHis tag were eliminated after digestion with Factor Xa and passing the peptide through a Ni-affinity column.

### Antimicrobial activity of RC-1

The disc diffusion susceptibility test was used to evaluate antimicrobial activity of the recombinant RC-1. The samples of recombinant RC-1 before and after refolding applications (100 μg/ml) were dropped onto a filter paper discs (0.1 mg/ml) and left to dry at room temperature. Then, the filters paper discs placed on an *E. coli*-inoculated Mueller-Hinton agar and a *Saccharomyces cerevisiae* inoculated yeast extract peptone dextrose (YPD) agar plates. Both *E. coli* and yeast inoculated plates were inoculated for 16 hrs at 35°C and 30°C, respectively.

### Protease assay

The bioassay used in this study was published by Rothan and co-workers [23]. In brief, endpoint reaction mixtures with total volume of 200 μl were prepared consisted of 20 μM fluorogenic peptide substrate (Boc-Gly-Arg-Arg-AMC), 2 μM of recombinant NS2B-NS3pro, with or without RC-1 of varying concentrations, buffered at pH 8.5 with 200 mM Tris–HCl. The RC-1 was initially prepared in Tris–HCl buffer and assayed at five different concentrations, ranging from 9.3 - 150 μM. Three types of reactions which are buffer, buffer with enzyme and buffer with enzyme and different concentrations of RC-1were incubated at 28°C, 37°C and 40°C for 30 minutes. Subsequently, the substrate was added to each reaction mixture and incubated at same temperatures for another 30 minutes. Measurements were performed in triplicates using Tecan Infinite M200 Pro fluorescence spectrophotometer. Substrate cleavage was normalized against buffer only (control) and optimized at the emission at 440 nm upon excitation at 350 nm. The readings were then used for calculating *K*_*m*_ values of peptide substrate and IC_50_ values of peptide inhibitors using non-linear regression models in GraphPad Prism 5.0 software as previously described [24].

### Maximum non-toxic dose test (MNTD)

Vero cells were seeded at 1 × 10^4^ cells per well in triplicate in 96 well plates and propagated at optimal conditions (37°C, 5% CO_2_ in humidified incubator). Recombinant RC-1 was diluted to serial concentrations (37.5, 75, 100, 125, 150 and 175 μM) with DMEM media supplemented with 2% FBS. Each dilution was tested in triplicate. Two control wells were included in each experiment: culture medium without RC-1 and culture medium with different concentrations of the RC-1 in the absence of cells to subtract the background value of RC-1 in the culture medium. The cell cultures were analyzed at 24, 48 and 72 hours using Non-Radioactive Cell Proliferation assay (Promega, USA) according to the manufacturer’s instructions.

### Treatment of DENV-2 infected cells with recombinant RC-1

Three independent experiments were carried out for pre, simultaneous and post-infection treatments with RC-1 at 24, 48 and 72 hrs of each infection in triplicates. Vero cells were grown in a 24-well tissue culture plate (1 × 10^5^ cells/well), incubated 24 hrs under optimal conditions and infected with DENV-2 (MOI of 2). For pre-treatment infection, 150 μM of RC-1 was added to the cells before virus inoculation and incubated for 24 hrs. Afterwards, DENV-2 supernatant was added, followed by incubation for 1 hr with gentle shaking every 10 min for optimal virus to cells contact. The virus supernatant was removed, and the cells were washed twice with fresh serum-free DMEM media to remove residual virus. New complete DMEM media were added and the cultures were incubated for 24, 48 and 72 hrs at 37°C, supplemented with 5% CO_2_. Same applications were used for simultaneous treatment infection except the peptide was mixed with the virus supernatant and incubated at 37°C for 1 h, and then inoculated onto Vero cells. The post-treatment infection was carried out after inoculation of Vero cells with DENV-2 and the complete DMEM medium was added containing the RC-1. Then, the cultures were incubated for 24, 48 and 72 hrs at 37°C and 5% CO_2_ with three wells of infected cells in each experiment were kept without RC-1 as controls. Cellular supernatants were collected and stored at −80°C for viral quantification using real time PCR.

### Dengue virus quantification by Real time PCR

For quantification of copy number of DENV-2 RNA, the standard curve was generated by 10-fold serial dilution of known copies of DENV-2 RNA. Viral RNA was extracted from culture supernatant using QIAmp viral RNA mini kit (Qiagen, Germany) according to the manufacturer’s instructions. A fragment located at the 5'UTR region of the virus genome was used to generate the primers. One-step RT-PCR using SyBr Green Master Kit (Qiagen, Germany) was used to conduct absolute quantification using ABI7300 machine from Applied Biosystems (Foster City, CA). The PCR profile was 1 cycle of 50°C for 2 min, 1 cycle of 95°C for 10 min, 40 cycles of 10 sec denaturation at 95°C, 20 sec annealing at 57.7°C with a single fluorescence emission measurement and 30 sec extension at 72°C, followed by 5 min at 72°C for final extension. Dissociation curve analysis was added to the end of each run. Results were analyzed using the company's Sequence Detection Software Version 1.3.

### Statistical analysis

All assays (Protease assay, Cytotoxicity assay, quantification of viral RNA by RT-qPCR) were done in triplicates. All statistical analyses were performed using GraphPad Prism version 5.01 (GraphPad Software, San Diego, CA). *P* values < 0.05 were considered significant. Error bars are expressed as ± SD.

## Results

### Production of recombinant RC-1 and NS2B-NS3pro in *E. coli*

**Figure 1:**
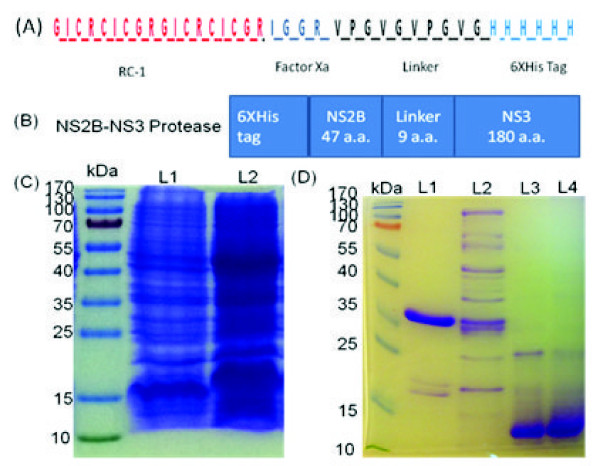
**Production of recombinant RC-1 and dengue NS2B-NS3pro by *E. coli. *** The sequence of RC-1 was linked by 10 amino acids to a 6XHis tag in pET 303/CT His vector. Factor Xa recognition sequence was inserted to cleave both the linker and 6XHis tag after purification. (**A**). The NS2B unit of dengue protease was linked to the NS3 by 9 amino acids and the entire DNA fragment was cloned into pQE30 vector downstream of a 6XHis tag (**B**). The recombinant *E. coli* were cultured in Luria-Bertani liquid medium and induced with Isopropylthio-β-D-galactoside (IPTG) to produce recombinant RC-1 (**C**, L1 before induction and L2 after induction) and the same procedure was applied to produce recombinant NS2B-NS3pro. The recombinant RC-1 and NS2B-NS3pro were purified by Ni-affinity column (**D**, L1 purified dengue protease, L2 NS2B-NS3pro before purification, L3 purified RC-1 and L4 purified RC-1 after refolding).

**Figure 2:**
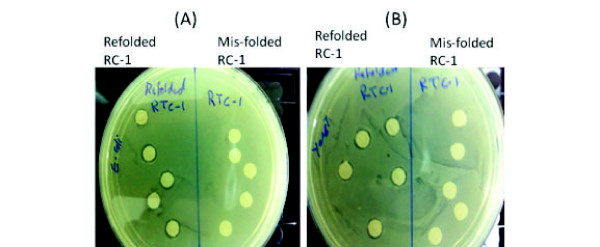
**Antimicrobial activity of recombinant RC-1 after refolding.** Each sample of RC-1 before and after refolding (0.1 mg/ml) was dropped on filter papers discs and left to dry at room temperature. The dry filter paper discs were placed on *E. coli* and yeast-inoculated antibiotic-free Mueller-Hinton-gar and YPD-agar plates respectively and incubated overnight at 37°C (*E. coli*) or 30°C (yeast). Recombinant RC-1 exhibited antimicrobial activity against *E. coli* (**A**) and yeast (*Saccharomyces cerevisiae*) (**B**) compared to mis-folded form and control plates (column buffer) as exhibited by the presence of a clear circular zone in the agar plate.

Our aim was to produce RC-1 in recombinant form in *E. coli* as a cost-effective expression system*.* In general, short peptides can be produced in a soluble form in *E. coli*. However, it is often challenging to have correct disulphide bond formation in cysteine rich peptides. We have produced RC-1 as a soluble recombinant peptide fused with ten amino acids linker followed by a 6XHis tag (Figure 1A). However, the product was biologically inactive due to the peptide mis-folding. The main challenge was to regenerate the activity of recombinant RC-1 with correct refolding of the peptide by appropriate reformation of the three disulphide bonds (Cys3-Cys16, Cys5-Cys14 and Cys7-Cys12). Thus, this required the reduction of all of mis-formed disulphide bonds by the action of *β*-mercaptoethanol before the refolding was carried out. Subsequently, the diluted basic environment was applied to allow the formation of intra-molecules disulphide bonds and reduction in the formation of inter-molecules disulphide bonds. We used ten amino acids linker to avoid any interaction between the 6XHis tag and disulphide bonds. To test the activity of RC-1 in inhibiting the NS2B-NS3pro, we produced NS2B-NS3pro in *E. coli* as a single-chain peptide. Nine amino acids linker was used to join NS2B with NS3pro downstream of the 6XHis tag sequence (Figure 1B). The recombinant proteins were detected by SDS-PAGE and purified using Ni-affinity column (Figure 1C and D). To validate the functionality of the refolded RC-1, we tested for its antimicrobial activity against *E. coli* and yeast. It was found to exhibit antimicrobial activity compared to the inactive mis-folded form (Figure 2A and B).

### The inhibitory potential of RC-1 against Dengue protease

**Figure 3:**
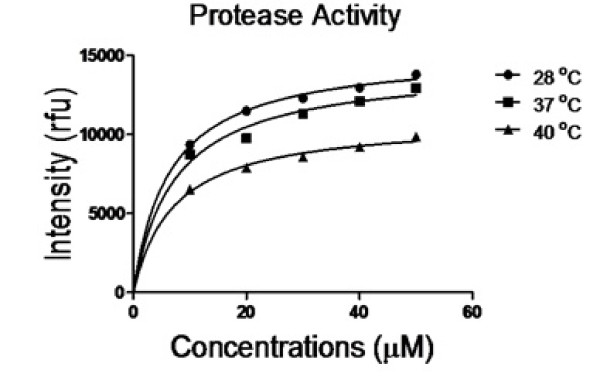
**The activity of dengue NS2B-NS3pro to cleave the fluorogenic peptide substrate at different temperature conditions.** The enzyme kinetic assay was carried out to evaluate dengue NS2B-NS3pro activity at temperature of mosquitoes (28°C), humans (37°C) and humans with severe dengue fever (40°C). Serial concentrations of the substrate peptide were used to assess the activity of recombinant DENV-2 protease. Triplicates were performed for all experiments and the relative fluorescence units (rfu) were measured using Tecan Infinite M200 Pro fluorescence spectrophotometer. Substrate cleavage was optimized at the emission at 440 nm upon excitation at 350 nm. After normalization of the results with the substrate incubated at same conditions, the highest activity of recombinant NS2B-NS3pro was observed at 28°C and lowest activity was at 40°C.

**Figure 4:**
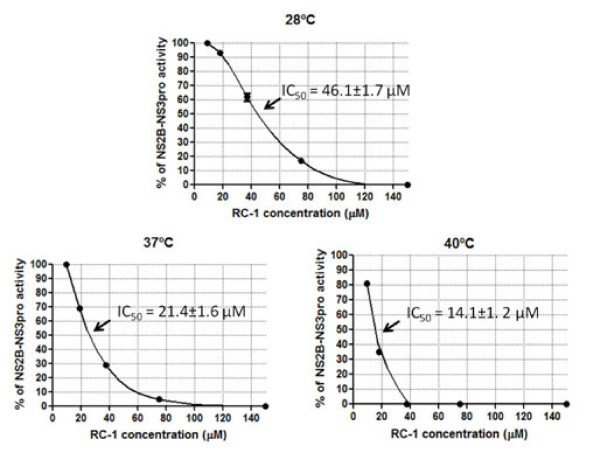
**The inhibition potential of RC-1 against the activity of DENV-2 NS2B-NS3pro.** End point protease inhibition assay was carried out to evaluate the inhibitory potential of RC-1 against the protease’s ability to cleave a fluorogenic peptide substrate (Boc-Gly-Arg-Arg-AMC). The reaction mixtures consisted of 20 μM substrate, 2 μM of enzyme, with or without RC-1 of varying concentrations incubated at 28°C, 37°C or 40°C for 30 minutes. The results were normalized with the substrate alone and substrate with protease incubated at same temperatures. The inhibition was found to be temperature dependent with the highest concentration (120 μM) at 28°C and the lowest concentration (37.5 μM ) at 40°C.

Dengue virus has the ability to propagate at different temperatures starting from mosquito temperature (28°C), normal human temperature (37°C) and at temperature of human with dengue fever (40°C). Therefore, we carefully studied the activity of NS2B-NS3pro at 28°C, 37°C and 40°C in the presence or absence of recombinant RC-1 peptide. The ability of NS2B-NS3pro to cleave the fluorogenic peptide substrate was variable at different temperatures. The highest activity was observed at 28°C and lowest activity was at 40°C while the activity at 37°C was in mid-range (Figure 3). The inhibition profile of RC-1 was variable at different temperatures (Figure 4). The results showed that at 28°C, RC-1 exhibited 100% inhibition of NS2B-NS3pro activity at the concentration of 120 μM. Similarly, a complete inhibition was observed at 100 μM and 37.5 μM at 37°C and at 40°C, respectively. The lowest 50% inhibitory concentration (IC_50_ value) of RC-1 was observed at 40°C (14.1 ± 1.2 μM) and the highest IC_50_ at 28°C (46.1 ± 1.7 μM) whilst at 37°C, the IC_50_ of RC-1 was 21.4 ± 1.6 μM.

### The inhibitory potential of RC-1 against dengue virus replication in Vero cells

**Figure 5:**
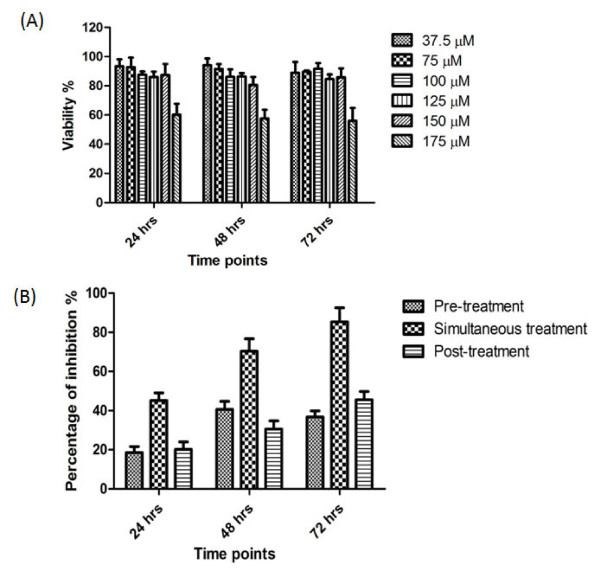
**The percentage of viral reduction in Vero cells after different treatments with RC-1.** (**A**) RC-1 was diluted to various concentrations (37.5, 75, 100, 125, 150 and 175 μM) with DMEM media supplemented with 2 % FBS and added to the cell culture. The viability of cells was analyzed at 24, 48 and 72 hrs using Non-Radioactive Cell Proliferation assay. (**B**) The cells were grown under the optimal conditions and infected with DENV-2 at the MOI of 2. The MNTD of RC-1 (150 μM) was used to treat infected Vero cells and the results were normalized with infected cells without RC-1 as controls. Pre, simultaneous and post-infection treatments with RC-1 were applied at 24, 48 and 72 hrs of each infection. The percentage of inhibition was the highest for simultaneous treatment compared to pre and post-treatment. (Two way ANOVA was used with Bonferroni post-test, P < 0.0001).

The maximum non-toxic dose (MNTD) of RC-1 concentration in Vero cells was found to be 150 μM for all of the three incubation periods, 24, 48 and 72 hrs (Figure 5A). This concentration of RC-1 was used in pre, simultaneous and post-infection treatment modes in Vero cells infected with DENV-2 at 24, 48 and 72 hrs. RNA of DENV-2 was extracted from RC-1 treated and control cells and quantified by real-time PCR. The results showed that the highest percentage of reduction in total viral RNA copies was observed at 24 hrs (45.5% ± 4.2), 48 hrs (70% ± 6.3) and 72 hrs (85% ± 7.1) for the simultaneous treatment mode compared to pre and post-treatment. Furthermore, there was a moderate reduction in viral replication at pre-treatments mode after 48 hrs (40%) and 72 hrs (38%) and post-treatment after 48 hrs (30%) and 72 hrs (45%) (Figure 5B).

## Discussion

We conducted this study to evaluate the effect of RC-1, a cationic cyclic peptide on the activity of dengue protease as a target to inhibit replication of dengue virus. The RC-1 was produced inexpensively as a soluble recombinant peptide in *E. coli* and the tri-disulphide bonds were correctly reformed in alkaline and diluted environment. It has been found that the activity of cyclic RC-1 is 3-fold more potent than the open chain analogue, indicating the disulphide bonds have a crucial role in its stability and functionality [19]. Furthermore, this peptide is able to provide long-lasting protection against viral infection since its analogues do not exhibit pro-inflammatory reactions, haemolytic activity or cellular toxicity [25,26]. Thus, the recombinant RC-1 peptide can be considered as a potential candidate for development of a successful drug to treat dengue and other infectious diseases.

The activity of recombinant NS2B-NS3pro was measured by a fluorescence-labelled substrate as previously described [23,27]. The function of dengue protease depends on the binding between NS2B and NS3pro to form NS2B-NS3pro complex. It has been found that this enzyme has an essential role in dengue virus replication through the cleavage of the viral polyprotein [12]. Therefore, blocking the active site or the interaction between the two subunits could directly disturb the activity of dengue protease and hence virus replication in host cells. Previous studies indicated that disulphide cyclic peptides have inhibition potential against dengue virus protease at different temperatures [15,16]. It has been known that the negatively-charged NS2B is positioned in a positive charged cleft on NS3pro [28]. Therefore it is possible that the RC-1, which is a cationic peptide, may inhibit the activity of dengue protease via direct interactions with the active site or by blocking the binding between the two subunits of the protease complex. Further studies are necessitated to elucidate the interaction between RC-1 and dengue protease that may have led to the inhibition of its activity.

Chemically synthesised RC-1 and its analogues by solid-phase peptide synthesis have been commonly used in many studies to determine its inhibitory potential against viral replication such as HIV [29], avian influenza H5N1 virus [30], Herpes Simplex Virus [31] and other microorganisms such as *Bacillus anthracis*[32] and *Staphylococcus aureus*[25]. However, to the best of our knowledge, there are no studies reported on the production of the RC-1 peptide in recombinant form in *E. coli*. Nonetheless, retrocyclins expression has been reported in other expression systems such as plant [33] and mammalian cells [30]. Unlike *E. coli*, these expression systems cannot be scaled up for larger production of recombinant protein in a cost-effective manner. We found that *E. coli* was able to produce soluble inactive recombinant RC-1 and the activity can be retrieved after refolding steps. The main challenge in maintaining the activity of recombinant RC-1 was in the reformation of the inter-molecular tri-disulphide bonds in its native order and to reduce intra-molecular disulphide bonds, which were addressed in this study. We have reported that the optimal refolding of cysteine rich proteins can be achieved in an alkaline and diluted environment in the presence of redox agents, which is in agreement with other studies [22]. The intra-molecular disulphide bonds were reduced after the dilution of the samples to 0.01 mg/ml in the presence of glycerol. In this study, the correct refolding was confirmed by the assessment of the RC-1 anti-microbial activity against *E.coli* and yeast (*Saccharomyces cerevisiae*).

Dengue protease exhibited highest activity at the temperature of mosquitoes (28°C) compared to the temperature of humans (37°C) and humans with severe dengue fever (40°C). Overall, this suggests that despite the low copy number of virus that is delivered by a mosquito bite, the viral virulence is very high. The complete inhibition of dengue protease had been achieved by different concentrations of RC-1 depending on the temperatures as expressed by various IC_50_ values. These are crucial factors to be taken into consideration in developing the RC-1 as an inhibitor-like drug in order to understand the drug stability and dose requirements at different stages of infection. In this study, the inhibition potential of RC-1 is temperature dependent (IC_50_ 21.4 μM at 37°C and 14.1 μM at 40°C) which is important to develop the RC-1 as an inhibitor-like drug to treat dengue. The inhibition potential of RC-1 is in the same range with other chemical compounds reported recently. These compounds inhibited recombinant dengue NS2B-NS3pro with IC_50_ of 15.4, 20.4 and 27.0 μM [34].

It has been known that the compound toxicity could hinder the development of drugs against dengue virus. In this study, recombinant RC-1 exhibited low cellular toxicity in Vero cells (150 μM) and has shown no noticeable cellular morphological abnormalities. In addition, it has been reported to have neither haemolytic activity nor cytotoxicity in different cell lines at up to a concentration of 500 μg/ml (250 μM) [26]. In addition, retrocyclins had no toxicity against human nasal epithelia at a concentration up to 200 μM, and did not induce any pro-inflammatory response in these cells [25]. In this study, the maximum non-toxic dose was used to treat the Vero cells infected with DENV-2. The viral replication was significantly declined after treatment with recombinant RC-1 at pre, simultaneous and post infection treatment modes. The highest significant reduction in dengue virus RNA replication was observed after simultaneous treatment of infected Vero cells. Therefore, it is possible that the recombinant RC-1 has an ability to reduce viral RNA replication by disturbing the activity of NS2B-NS3pro. Nevertheless, other possibilities of additional effects of RC-1 on Vero cells that may lead to reduction in viral RNA should be also taken in consideration.

## Conclusion

We successfully produced bioactive recombinant RC-1 in *E. coli* as a cost-effective expression system that can be developed for large-scale production. The recombinant RC-1 was able to inhibit dengue protease that led to reduction of dengue virus in Vero cells. These findings may have its importance in the development of therapeutic agent especially against dengue infection.

## Abbreviations

RC-1: Retrocyclin-1;DENV-2: Dengue virus serotype 2;NS2B: NS2B cofactor amino acids sequence 49–95 in DENV-2 NS2B and 1394–1440 in DENV-2 polyprotein;NS3pro: NS3 protease amino acids sequence 1–185 in NS3 protease and 1476–1660 in DENV-2 polyprotein;NS2B-NS3pro: NS2B fused to NS3pro via 9 amino acids (G4-T-G4);AMC: Fluorogenic peptide substrate (Boc-Gly-Arg-Arg-AMC).

## Competing interests

The authors declare that they have no competing interests.

## Authors’ contributions

HAR design performed the experiments and drafted the manuscript. HCH participated in the experiments and data analysis. TSR and SO participated in the analysis and preparation of the manuscript. NSR and RY participated in the design and drafted the manuscript. All authors approved the final manuscript.

## Pre-publication history

The pre-publication history for this paper can be accessed here:

http://www.biomedcentral.com/1471-2334/12/314/prepub
